# Characteristics of *BRCA1*/*2* mutations carriers including large genomic rearrangements in high risk breast cancer patients

**DOI:** 10.1007/s10549-017-4142-7

**Published:** 2017-02-15

**Authors:** Boyoung Park, Ji Yeon Sohn, Kyong-Ah Yoon, Keun Seok Lee, Eun Hae Cho, Myong Cheol Lim, Moon Jung Yang, Soo Jin Park, Moo Hyun Lee, See youn Lee, Yoon Jung Chang, Dong Ock Lee, Sun-Young Kong, Eun Sook Lee

**Affiliations:** 1 Graduate School of Cancer Science and Policy, National Cancer Center, 323 Ilsan-ro, Ilsandong-gu, Goyang-Si, Gyeonggi-do 10408 Korea; 2 Department of Laboratory Medicine, Center for Diagnostic Oncology, Hospital, National Cancer Center, 323 Ilsan-ro, Ilsandong-gu, Goyang-Si, Gyeonggi-do 10408 Korea; 3 Center for Breast Cancer, Hospital, National Cancer Center, 323 Ilsan-ro, Ilsandong-gu, Goyang-Si, Gyeonggi-do 10408 Korea; 4 Center for Uterine Cancer, Hospital, National Cancer Center, 323 Ilsan-ro, Ilsandong-gu, Goyang-Si, Gyeonggi-do 10408 Korea; 5 Gynecologic Cancer Branch, Research Institute, National Cancer Center, 323 Ilsan-ro, Ilsandong-gu, Goyang-Si, Gyeonggi-do 10408 Korea; 6 Translational Epidemiology Research Branch, Research Institute, National Cancer Center, 323 Ilsan-ro, Ilsandong-gu, Goyang-Si, Gyeonggi-do 10408 Korea; 7 Green Cross Genome, 314, Bojeong-dong, Giheung-gu, Yongin-si, Gyeonggi-do 446-713 Korea; 8 College of Veterinary Medicine, Konkuk University, 120 Neungdong-ro, Gwangjin-gu, Seoul, 05029 Korea; 9 Breast & Endocrine Cancer Branch, Research Institute, National Cancer Center, 323 Ilsan-ro, Ilsandong-gu, Goyang-Si, Gyeonggi-do 10408 Korea; 10 National Cancer Control Institute, National Cancer Center, 323 Ilsan-ro, Ilsandong-gu, Goyang-Si, Gyeonggi-do 10408 Korea; 11 Department of Surgery, Keimyung University School of Medicine, 56 Dalseong-Ro, Jung-Gu, Daegu, 700-712 Korea

**Keywords:** *BRCA1/2* mutation, Large genomic rearrangements, Breast cancer, Genetic counseling, Family counseling

## Abstract

**Purpose:**

We investigated the prevalence of *BRCA1/2* small mutations and large genomic rearrangements in high risk breast cancer patients who attended a genetic counseling clinic.

**Methods:**

In total 478 patients were assessed for *BRCA1/2* mutations by direct sequencing, of whom, 306 were identified as non-carriers of *BRCA1/2* mutation and assessed for large rearrangement mutations by multiplex ligation-dependent probe amplification. Family history and clinicopathological characteristics of patients were evaluated.

**Results:**

Sixty-three mutation carriers (13.2%) were identified with *BRCA1* mutations (6.3%) and *BRCA2* mutations (6.9%), respectively. Mutation frequency was affected by familial and personal factors. Breast cancer patients with family history of breast and ovarian cancer showed the highest prevalence of *BRCA1/2* mutations (67%), and triple-negative breast cancer (TNBC) patients showed high *BRCA1* mutation prevalence (25%). The three probands of *BRCA1* deletion (1%) represented both familial risk and personal or clinicopathological risk factors as two with TNBC and one with bilateral ovarian cancer.

**Discussion:**

This is the largest study assessing large genomic rearrangement prevalence in Korea and *BRCA1* deletion frequency was low as 1% in patients without *BRCA1/2* small mutations. For clinical utility of large genomic rearrangement testing needs further study.

## Introduction

Germline mutations in the *BRCA1/2* genes are the most important cause of hereditary breast cancer [1]. The average cumulative risk of breast cancer in female carriers above 70 years is estimated to be 57–65 and 45–49% for *BRCA1* and *BRCA2* mutation carriers, respectively [2, 3].

Risk of hereditary cancers is assessed by taking into account familial and personal factors or clinicopathological characteristics of cancers such as triple-negative breast cancers (TNBC), which do not express the genes for estrogen receptor (ER), progesterone receptor (PR), or HER2 receptor [4]. With this information, women with a high risk of developing hereditary cancers are recommended to be tested for mutations in *BRCA1/2* genes [5, 6]. Given that this test targets a high-risk population and the further management plans are largely dependent on the results of *BRCA1/2* mutation testing are important.

Direct Sanger sequencing, conformation-sensitive gel electrophoresis, and denaturing high-performance liquid chromatography have been used to identify *BRCA1/2* mutations. However, these techniques cannot identify large genomic rearrangements, which are often reported in patients with negative results from conventional direct sequencing [7, 8]. This may lead to an underestimation of mutation prevalence and provide false-negative information to patients and their families. Previous studies showed varying results with the prevalence of large genomic rearrangements ranging from 0 to 30% [9–14]. This wide range might be caused by different genetic backgrounds and the different inclusion criteria of various studies [11]. Thus, we investigated the prevalence of *BRCA1/2* large genomic rearrangements using multiplex ligation-dependent probe amplification in high-risk breast cancer patients with negative results for *BRCA1/2* mutation by direct sequencing.

## Materials and methods

### Study process

All the patients who were referred to a genetic counseling clinic and screened for *BRCA1/2* mutation, at the National Cancer Center between April 2008 and 2015, were initially considered for this study. Among the total 523 patients screened for *BRCA1/2* mutations, we excluded 28 family members of known *BRCA1/2* mutation carriers and 17 patients with other types of cancers. There were 478 probands with breast cancer included in this study. The referred criteria for breast cancer were: (1) breast cancer patients with a family history of breast or ovarian cancer; (2) breast cancer patients who were 40 years or younger during diagnosis, who had bilateral breast cancer or breast cancer with other primary malignancy, or male breast cancer patients, in accordance with the standard of National Medical Insurance Reimbursement in Korea. In the genetic counseling process, all patients’ mutation probabilities were estimated by CaGene5.0 software [15] using pedigree information up to second-degree relatives and considering estimates by BRCAPRO [16] and Myriad [17]. Clinicopathological characteristics of cancer, such as the stage and the hormone receptor and human epidermal growth factor receptor 2 (HER2) status, were evaluated by reviewing medical records.

**Fig. 1:**
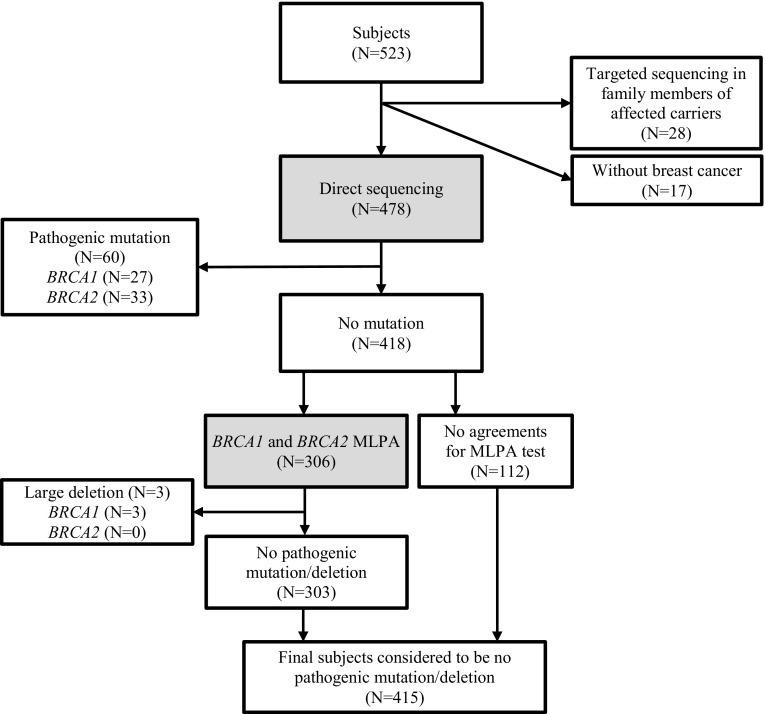
The study flowchart outlining the number of subjects and the genetic testing approach used in the study. A total of 478 breast cancer patients were included and multiple ligation-dependent probe amplification (MLPA) analysis was performed for 306 patients who did not have small mutations in the *BRCA1 and BRCA2* genes and agreed for this study

The initial *BRCA1/2* mutation test was performed by PCR amplification and direct sequencing covering all exons and flanking intronic sequences. A subset (73%) of the 418 cases designated as non-carriers by direct sequencing, agreed to participate in the study. Written informed consent was obtained from 306 patients, and they were screened for the presence of large genomic rearrangements using a multiplex ligation-dependent probe amplification (MLPA) assay. This study protocol was approved by the institutional review board of the National Cancer Center (NCC2015-0177). The selection of patients and the test process is presented in the Fig. 1.

### Direct sequencing for mutation detection of *BRCA1* and *BRCA2*, and nomenclature

Genomic DNA was extracted from peripheral blood using the Chemagic DNA Blood 200 Kit (Chemagen, Baesweiler, Germany). Amplified products were sequenced on an ABI 3500xl analyzer (Applied Biosystems, Foster City, CA, USA), using the Bigdye Terminator v3.1 Cycle Sequencing Kit. Sequences were analyzed using Sequencher v4.10.1 software. The clinical significance of each sequence variation was determined according to the Breast Cancer Information Core database (BIC: http://research.nhgri.nih.gov/bic/) and the recommendations of the American College of Medical Genetics [18]. All the mutations are described according to HUGO-approved systematic nomenclature (http://www.hgvs.org/mutnomen/). GenBank accession sequences NM_007294.3 and NM_000059.3 were used as reference sequences for *BRCA1* and *BRCA2*, respectively. Traditional mutation nomenclatures of the BIC were used for description.

### Multiplex ligation-dependent probe amplification

MLPA was performed to detect large genomic rearrangements using the SALSA P002-D1 *BRCA1* Kit (MRC Holland, Amsterdam, Holland) for *BRCA1* and P045-B3 *BRCA2* Kit (MRC Holland, Amsterdam, Holland) for *BRCA2*. PCR products were analyzed using an ABI 3500xl analyzer with GeneMarker v2.4.0 demonstration program (Softgenetics, State College, PA, USA). Peak heights were normalized, and a deletion or duplication was identified when the normalized peak ratio value was below 0.75 or above 1.30, respectively.

### Risk factors and statistical analysis

The subjects were classified according to familial and personal factors. Familial factors taken into account included family history of breast cancer, number of family members with breast cancer, closest degree of family members with breast cancer, and family history of ovarian cancer. Forty-two of the 306 patients had a family history of ovarian cancer. Personal factors taken into account included early onset of breast cancer which is defined as the development of breast cancer before the age of 40, bilateral breast cancer irrespective of age at onset, both breast and ovarian cancer irrespective of age at onset, multiple organ cancers defined as breast cancer patients with other primary organ cancer except ovarian cancer, and male cancer. The clinicopathological factors considered were age at diagnosis of breast cancer, stage, and the hormone receptor (including estrogen and PR) and HER2 status.

The frequencies of mutations were presented according to familial and personal factors and clinicopathological characteristics. All analyses were conducted using SAS 9.2 software (SAS Institute, Cary, NC).

## Results

### Mutational status according to subjects’ characteristics

Pathogenic mutations in *BRCA1/2* genes, including large genomic rearrangements, were detected in 63 of 478 (13.2%) patients. In total, 30 *BRCA1* mutation carriers (6.3%) and 33 *BRCA2* mutation carriers (6.9%) were identified. Table 1 shows the frequency of *BRCA1/2* mutations according to familial and personal factors, and clinicopathological characteristics. *BRCA1/2* mutation prevalence in familial breast cancer cases was 15.9%, which was significantly higher in non-familial breast cancer cases (6.0%, *P* = 0.004). When personal factors were considered, *BRCA1/2* mutations were observed in 11.6% of early-onset breast cancer patients, 14.9% of bilateral breast cancer patients, and 66.7% of patients who were diagnosed with both breast and ovarian cancer. The prevalence of *BRCA1/2* mutations also differed significantly according to hormone receptor and HER2 status (*P* = 0.003 and <0.001, respectively). The prevalence of *BRCA1/2* mutation according to the combinations of familial and personal factors with denominators and numerators is described in Appendix Table 4.

**Table 1:** The frequencies of *BRCA1 and BRCA2* mutations^a^ in high-risk breast cancer patients according to familial and personal risk factors (*N* = 478)

Risk category	Total	*BRCA1* mutation	*BRCA2* mutation	*BRCA1/2* mutation
	*N* (%^d^)	*N* (%^e^)	*N* (%^e^)	*N* (%^e^)
*Family history*				
Breast cancer family only (without ovarian cancer)^§^	303 (63.4)	14 (4.6)	29 (9.6)	43 (14.2)
1 breast cancer family	253 (52.9)	11 (4.4)	17 (6.7)	28 (11.1)
2 ≤breast cancer families*^,§^	50 (10.5)	3 (6.0)	12 (24.0)	15 (30.0)
Breast cancer families in 1st degree relatives^b^	217 (45.4)	13 (6.0)	20 (9.2)	33 (15.2)
Breast cancer families in second/third degree relatives^b,§^	86 (18.0)	1 (1.2)	9 (10.5)	10 (11.6)
Ovarian cancer family^b^				
Without breast cancer^§^	29 (6.1)	6 (20.7)	0 (0.0)	6 (20.7)
With breast cancer*	13 (2.7)	4 (30.8)	2 (15.4)	6 (46.2)
Any of breast/ovarian cancer families^c,^*	345 (72.2)	24 (7.0)	31 (9.0)	55 (15.9)
No family history*	133 (27.8)	6 (4.5)	2 (1.5)	8 (6.0)
*Personal history*				
Early-onset breast cancer (age < 40)	199 (41.6)	12 (6.0)	11 (5.5)	23 (11.6)
Bilateral breast cancer	47 (9.8)	2 (4.3)	5 (10.6)	7 (14.9)
Multiple organ cancers^f^	27 (5.6)	0 (0.0)	2 (7.4)	2 (7.4)
Both breast and ovarian cancer*	6 (1.3)	3 (50.0)	1 (16.7)	4 (66.7)
Male breast cancer	4 (0.8)	0 (0.0)	0 (0.0)	0 (0.0)
*Clinicopathological factor*				
Age at diagnosis				
<40	186 (38.9)	12 (6.5)	11 (6.8)	23 (14.3)
40–49	172 (36.0)	10 (5.8)	15 (8.7)	25 (14.5)
50–59	89 (18.6)	7 (7.9)	6 (6.7)	13 (14.6)
60–79	31 (6.5)	1 (3.3)	1 (3.3)	2 (6.5)
Stage*				
0	55 (11.5)	0 (0.0)	0 (0.0)	0 (0.0)
I	173 (36.2)	12 (6.9)	12 (6.9)	24 (13.9)
II	159 (33.3)	12 (7.6)	18 (11.3)	30 (18.9)
III+	88 (18.4)	5 (7.4)	2 (2.9)	7 (10.3)
Unknown	3 (0.6)	0 (0.0)	0 (0.0)	0 (0.0)
Hormone receptor status*^,§^				
ER+ & PR+	307 (64.2)	5 (1.6)	25 (8.1)	20 (9.8)
ER+ & PR−	44 (9.2)	1 (2.3)	4 (9.1)	5 (11.4)
ER− & PR+	7 (1.5)	1 (14.3)	0 (0.0)	1 (14.3)
ER− & PR−	115 (24.1)	23 (20.0)	4 (3.5)	27 (23.5)
Unknown	5 (1.0)	0 (0.0)	0 (0.0)	0 (0.0)
Subtype according to hormone receptor and HER2 status*^,§^				
HR+ & HER2−	252 (52.7)	6 (2.4)	19 (7.5)	25 (9.9)
HR− & HER2+	28 (5.9)	1 (3.6)	0 (0.0)	1 (3.6)
HR+ & HER2+	37 (7.7)	0 (0.0)	2 (5.4)	2 (5.4)
Triple-negative	76 (15.9)	19 (25.0)	3 (4.0)	22 (29.0)
Unclassifiable	85 (17.8)	4 (4.7)	9 (10.6)	13 (15.3)
Total	478 (100.0)	30 (6.3)	33 (6.9)	63 (13.2)

*HR* hormone receptor, *HER2* human epidermal growth factor receptor 2

* P value <0.05 for *BRCA1/2* mutation prevalence between those included in each category and those not

^§^ *P* value <0.05 between *BRCA1* and *BRCA2* ratio in carriers

^a^Including three patients with large genomic rearrangements in BRCA1 gene

^b^Among 42 patients who had family history of ovarian cancer, 40 had one family member with ovarian cancer history and 2 had two family members with ovarian cancer history

^c^Closest degree of relatives with breast cancer

^d^Percent among all subjects (column percent)

^e^Percent among subjects with each risk category (row percent)

^f^Multiple organ cancer was defined as breast cancer patients with other primary organ cancer except ovarian cancer

### Patients with large genomic rearrangements

**Fig. 2:**
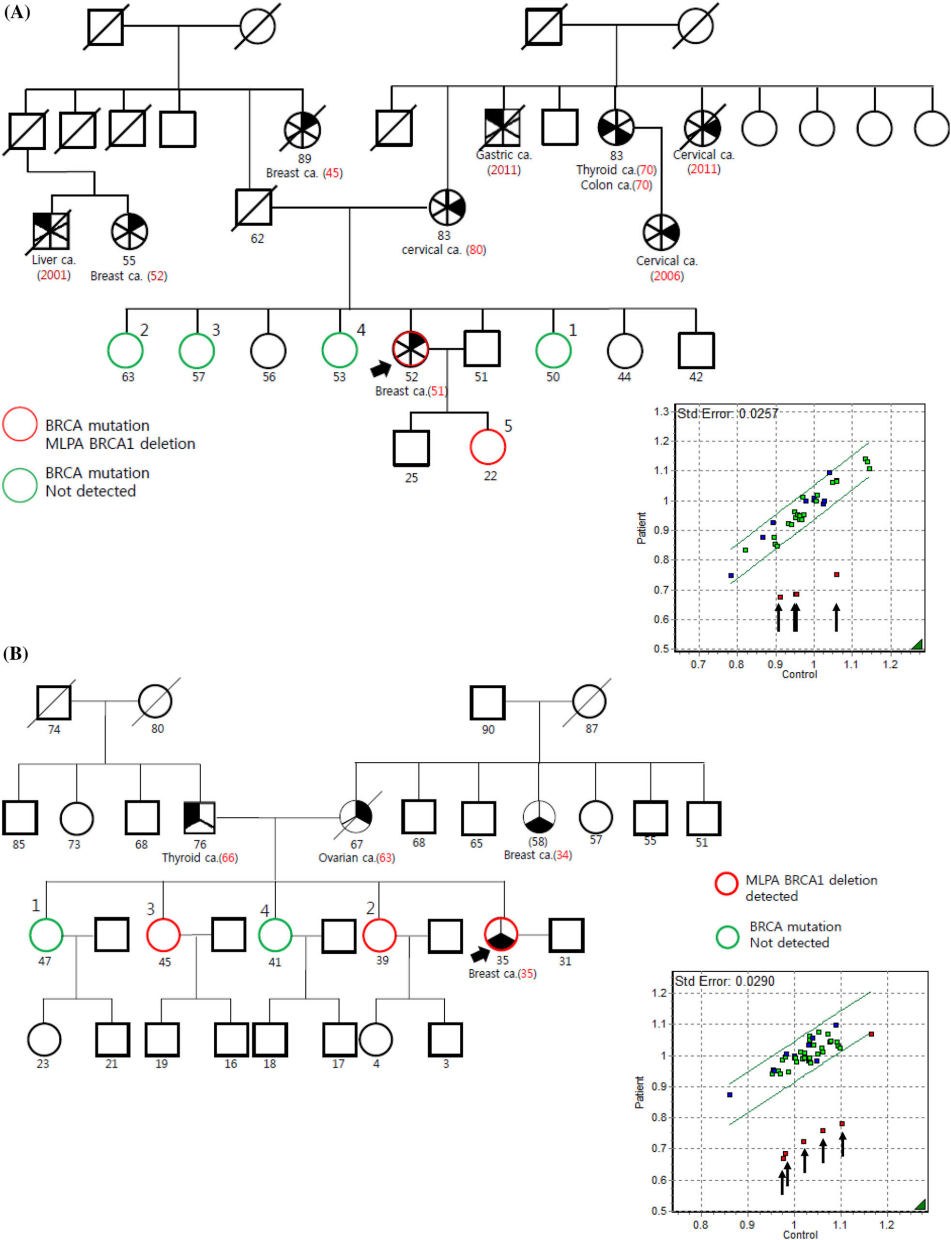

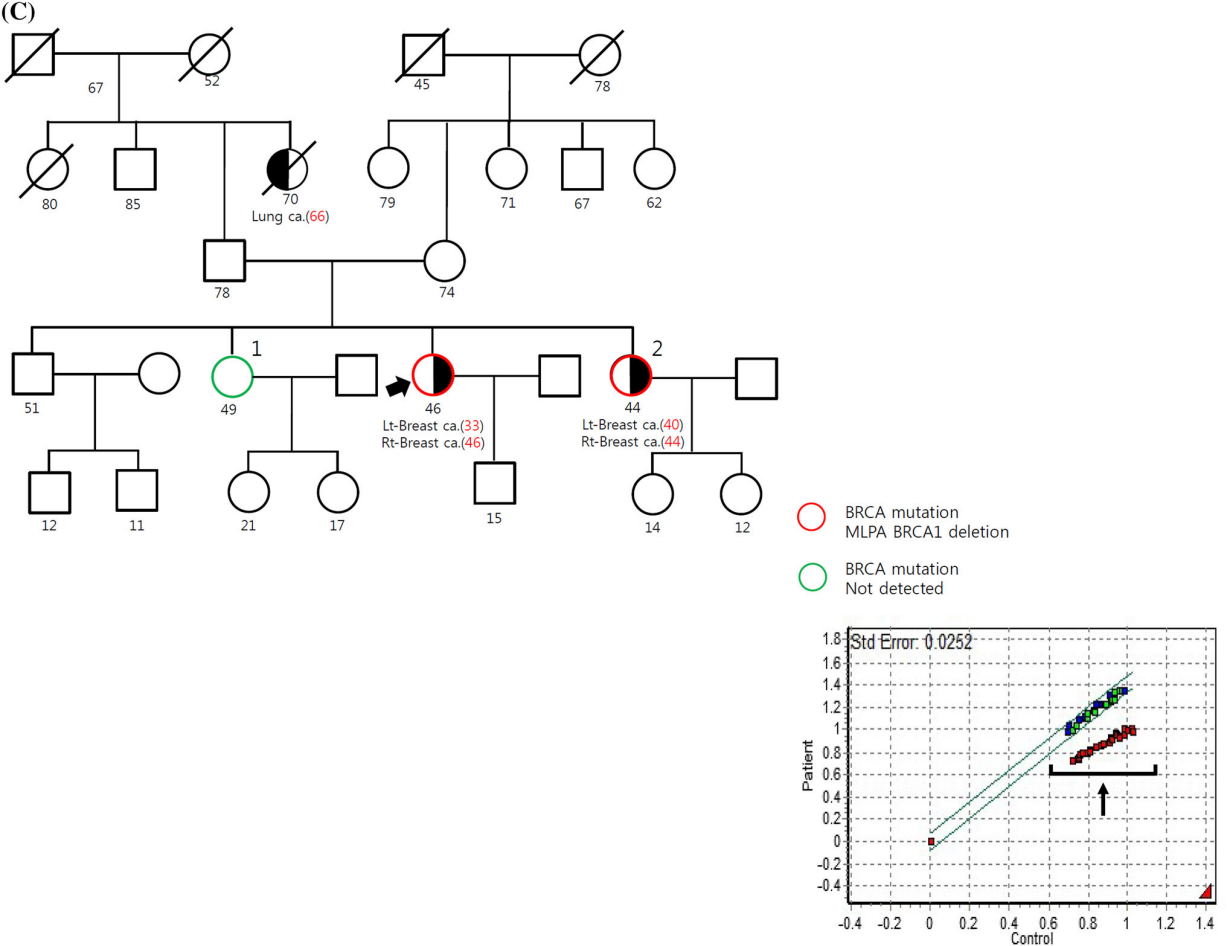
The 3 *BRCA1* LGRs identified in the study using MLPA screening. The MLPA analysis demonstrates **a** exons 5–8 deletion, **b** exons 22–24 deletion, and **c** exons 1–14 deletion. Exons having a reduced peak ratio are denoted with the *arrows*

Three *BRCA1* deletion carriers were identified by MLPA from 306 patients *BRCA1/2* mutation negative by standard sequencing. These deletion mutations account for 10% of all *BRCA1* mutation carriers (Fig. 1). The characteristics of each patient are summarized in Table 2 and detailed explanation is as following. Patient A, diagnosed with ductal carcinoma in situ with TNBC at the age of 51, carried exons 5–8 deletion (Fig. 2a). She had a second- and a third-degree relative with breast cancer. Estimated mutation probabilities for *BRCA1/2* before mutation test were 0.8% by BRCAPRO and 5.3% by Myriad. Following genetic counseling about the *BRCA1* deletion, genetic testing was performed on her daughter and three sisters without breast cancer and her daughter was found to have the same deletion.

**Table 2:** Characteristics of the probands with *BRCA1* large genomic rearrangements

	Personal factor				Familial factor			Mutation carrier risk
Pt	Early-onset BC (age at diagnosis)	Bilateral BC	Both BC and OC	TNBC	Family history of BC (number, closest degree)	Family history of OC (number, closest degree)	Other cancer	BRCAPRO/Myriad
A	No (51)	No	No	Yes	Yes (2, second degree)	No	Liver Cervix Stomach Thyroid Colon	0.8/5.3
B	Yes (35)	No	No	Yes	Yes (1, second degree)	Yes (1, 1st degree)	Thyroid	57.2/39.2
C	Yes (33)	Yes	Yes	No	Yes (1, first degree)	No	Lung	51.2/15.8

Patient B, diagnosed with stage III TNBC at the age of 35, carried exons 22–24 deletion (Fig. 2b). The patient had a first-degree relative with ovarian cancer and a second-degree relative with breast cancer. Estimated mutation probabilities for *BRCA1/2* mutation test were 57.2% by BRCAPRO and 39.2% by Myriad. Genetic testing was performed on her four sisters without breast cancer and of them, two sisters were found to have the deletion.

Patient C, was diagnosed with bilateral stage I breast cancer for the first time at age 33 and for the second time at age 46. She was not a TNBC case, and carried a *BRCA1* gene lacking exons 1–14 (Fig. 2c). The estimated mutation probabilities for *BRCA1/2* mutation test were 51.2% by BRCAPRO and 15.8% by Myriad. After enrollment in this study, she was newly diagnosed with ovarian cancer. Genetic testing was performed on her two sisters and one of her sisters, diagnosed before with bilateral breast cancer, carried a *BRCA1* gene with the same large genomic rearrangements.

### Pathogenic variants of *BRCA1/2* genes found in this study

The pathogenic variants of *BRCA1/2* genes found in this study are presented in Table 3. Overall, 24 pathogenic variants in *BRCA1* gene and 19 in *BRCA2* gene were found in 63 index cases mutations. Many of these mutations have been reported in the BIC or previous studies in Korea. The most frequent mutation was c.7480C>T in *BRCA2* and it was found in nine patients (14.3%). The next most frequent mutations were c.1399A>T in *BRCA2* gene and c.390C>A in *BRCA1* gene, which were found in four and three patients, respectively. The large genomic rearrangements found through MLPA were located in *BRCA1* gene, including an exon 1–14 deletion, exon 5–8 deletion, and exon 22–24 deletion.

**Table 3:** Frequency of pathogenic variants of BRCA*1* and BRCA*2* genes in patients

Gene	Exon/intron	BIC nomenclature	HGVS cDNA	HGVS protein	*N*
*BRCA1*	IVS5	IVS5+1G>A	c.212+1G>A	–	1
	7	509C>A	c.390C>A	p.Tyr130*	3
	11	1041_1042delAG	c.922_923delAG	p.Ser308Glufs*	1
	11		c.922_924delAGCinsT	p.Ser308*	1
	11	1137delG	c.1018delG	p.Val340Glyfs*	1
	11	1599C>T	c.1480C>T	p.Gln494*	1
	11		c.14923_1494delTC	p.Leu498Hisfs*	1
	11	1630dupG	c.1511dupG	p.Lys505*	1
	11		c.1516delA	p.Arg506Glysfs*	2
	11		c.2354T>A	p.Leu785*	1
	11	3415delC	c.3296delC	p.Pro1099Leufs*	1
	11	3746dupA	c.3627dupA	p.Leu1210Glufs*	1
	11	3819del5	c.3700_3704delGTAAA	p.Val1210Aspfs*	1
			c.4110C>T	p.Glu1331*	1
	16	5100G>T	c.4981G>T	p.Glu1661*	2
	20	5379G>T	c.5260G>T	p.Glu1754*	2
	IVS21		c.5332+4delA	–	1
	23		c.5445G>A	p.Trp1815*	2
	IVS23	IVS23+1G	c.5467+1G>A	–	1
	24	5602delG	c.5483delG	p.Cys1828Leufs*	1
	24	5615del11insA	c.5496_5506delGGTGACCCGAGinsA	p.Val1833Serfs*	1
	1–14	Exon 1–14 deletion			1
	5–8	Exon 5–8 deletion			1
	22–24	Exon 22–24 deletion			1
*BRCA2*	7	173G>T	c.518G>T	p.Gly173Val	1
	9	983del4	c.755_758delACAG	p.Asp252Serfs*	2
	10	1222delA	c.994delA	p.Ile332Phefs*	1
	10	1627A>T	c.1399A>T	p.Lys467*	4
	11	3026delCA	c.2798_2799delCA	p.Thr933Argfs*	1
	11		c.3096_3110delAGATATTGAAGAAC	p.Asp1033Ilefs*	1
	11	3972del4	c.3744_3747delTGAC	p.Ser1248Glufs*	3
	11	6019C>T	c.5791C>T	p.Gln1931*	1
	11	6781delG	c.6553delG	p.Ala2185Leufs*	1
	14		c.7258G>T	p.Glu2420*	2
	15	7708C>T	c.7480C>T	p.Arg2494*	9
	15		c.7486G>T	p.Glu2420*	1
	18		c.8300_8301insAC	p.Pro2767Hisfs*	1
	22	9179C>G	c.8951C>G	p.Ser2984*	1
	23	9304C>T	c.9076C>T	p.Gln3026*	1
	24		c.9253delA	p.Thr3085Glnfs*	1
	25	9503del2	c.9275_9276delAT	p.Tyr3092Phefs*	1
	25	9641T>G	c.9413T>G	p.Leu3138*	1

## Discussion

Here, we detected *BRCA1/2* mutations in 13.2% of high-risk breast cancer patients who were referred to a genetic counseling center. Of the *BRCA1/2* carriers, 5% (3 out of 63) were identified by MLPA after negative direct sequencing results. All the large genomic rearrangements were found in *BRCA1* gene. Therefore, 10% of *BRCA1* carriers (3 out of 30) would have not been identified if MLPA had not been conducted. In Korea, *BRCA1/2* mutation screening has been covered by National Health Insurance since 2012 for those who meet certain criteria. Because more than 95% of this study population was recruited after 2012, our results could provide a more current measure of the prevalence of *BRCA1/2* mutations.

The prevalence in familial breast cancer cases was 15.9% in this study, which was slightly lower than previous results from Korea, which ranged 19.4–30.0% [19–22], and results from Western countries [23]. Mutation prevalence according to each personal factor in this study was comparable with previous Korean studies [19, 21, 22, 24, 25]. TNBC is an important factor used to select breast cancer patients for *BRCA1/2* mutation testing [5] and this was confirmed in our study. Considering that the mutation prevalence varies according to overlapped risk factors (Appendix Table 4), multiple combinations of familial or personal factors need to be considered for a more detailed risk assessment.

The prevalence of large genomic rearrangement after negative direct sequencing results in previous studies targeting potential hereditary cancer subjects in diverse ethnic groups ranged 0–5%, and was particularly low in Asian countries [12, 26–33] (Appendix Table 5). With the exception of two studies, most large genomic rearrangements have been identified in the *BRCA1* gene [12, 28], and all of the large genomic rearrangements identified in Korea [9, 10], including in this study, were in the *BRCA1* gene. The common characteristics of seven *BRCA1* large genomic rearrangement cases were that they all have a family history of breast and/or ovarian cancer with at least one additional personal factor. These personal factors included bilateral breast cancer, young age at onset (≤40 years old), both breast and ovarian cancer, and TNBC. Therefore, at least for *BRCA1*, the MLPA test should be considered for breast cancer patients with a family history of breast and/or ovarian cancer and additional personal factors such as bilateral breast cancer, young age at onset, and TNBC during the genetic counseling process. In our study population with large genomic rearrangements, the compliance with the prophylactic strategies was quite good (Appendix Table 6). 

Several limitations of this study should be mentioned. Firstly, this study was performed by the patients of a single institute. Secondly, we could not conduct MLPA test for all *BRCA1/2* small mutation non-carriers due to patient non-participation, suggesting possible participation bias. However, this study was designed to reflect common clinical settings, following the patients in the process of genetic counseling. Thirdly, the detected large genomic rearrangements through MLPA test were not confirmed by different MLPA probes or other platforms. However, the three detected large genomic rearrangements were multi-exon deletions and detected in multiple family members, showing apparent inheritance patterns, suggesting minimum probabilities of false positives. Fourth, the family history was obtained by proband recollection and we did not consider the validity and reliability of the information.

In conclusion, the prevalence of *BRCA1/2* mutations was dependent on familial and personal factors. Subjects with both familial and personal factors had a much higher risk of carrying *BRCA1/2* mutations. The MLPA test for BRCA1 mutation could be recommended for breast cancer patients with a family member with breast and/or ovarian cancer and additional personal factors, and who tested negative for *BRCA1/2* small mutations in initial testing.

## Appendix

See Tables 4, 5, and 6.

**Table 4:** The frequencies of *BRCA1 and BRCA2* mutations according to combined family and personal characteristics

^a^Multiple organ cancer was defined as breast cancer patients with other primary organ cancer except ovarian cancer

**Table 5:** The prevalence of BRCA*1*/*2* mutation and large genomic rearrangements in subjects without small mutation from direct sequencing

Country	Number of subject	Total prevalence (%)		Prevalence among subjects with negative result of direct sequencing (%)	
		BRCA*1*	BRCA*2*	LRG in BRCA*1*	LRG in BRCA*2*
Hong Kong [24]	1236	4.6	5.1	0.4	0.3
Poland [27]	281	28.8	–	1.5	–
Lebanese Arab [22]	250	2.8	2.8	0.0	0.0
Mexico [29]	188	18.0	3.2	2.0	0.0
Slovakia [23]	585	14.5	11.5	5.0	0.0
Malaysia [11]	324	7.4	7.1	0.6^a^	0.3^a^
Malaysia [28]	100	–	–	2.0^a^	0.0
Poland [26]	64	67		4.6	0.0
Singapore [25]	100	–	–	2.0	1.0
Korea [8]	221	35.3		2.1	0.0
Korea [9]	122	–	–	0.8	0.0
Korea—this study	478	6.3	6.9	1.0	0.0

^a^Not certain whether the prevalence is from whole subject or from limited subjects with *BRCA1/2* mutation negative

**Table 6:** Follow-up status of the family members with multiple ligation-dependent probe amplification (MLPA) positive result from family with *BRCA1* large genomic rearrangements

Pt	Relationship (age)	Interval between proband identification and first genetic counseling	Known comorbidities at first genetic counseling	Interval between MLPA (+) to first preventive screening	Result of initial preventive screening for ovarian cancer	Result of initial preventive screening for breast cancer	Conducted prophylactic therapy and family care	Future plan for prophylaxis and family care
A	Daughter (23)	5 months	None	4 days	None	None	Oral contraceptive	Annual breast cancer screening until the age 25 Ovarian cancer screening per 3 months Recommended family screening for brother (age 25)
B	Sister 1 (39)	4 months	Thyroid disease	13 days	None	None	BSO (3 months after identifying carrier)	
	Sister 2 (45)	4 months	None	13 days	None	None	BSO (3 months after identifying carrier)	Recommended family screening for 2 sons in the future (age 19 and 16)
C	Sister (44)	1 months	Left breast cancer (TNBC, age 40) Right breast cancer (HR positive, age 44)	1 day	None	Known breast cancer	–	BSO Recommended family screening for 2 daughters in the future (age 15 and 13)

*BSO* bilateral salpingo-oophorectomy, *TNBC* triple-negative breast cancer, *HR* hormone receptor
